# Application of clinical pathway using electronic medical record system in pediatric patients with supracondylar fracture of the humerus: a before and after comparative study

**DOI:** 10.1186/1472-6947-13-87

**Published:** 2013-08-11

**Authors:** Ki Hyuk Sung, Chin Youb Chung, Kyoung Min Lee, Seung Yeol Lee, Soyeon Ahn, Somin Park, In Ho Choi, Tae-Joon Cho, Won Joon Yoo, Jung Hyun Lee, Moon Seok Park

**Affiliations:** 1 Department of Orthopaedic Surgery, Kwandong University Myongji Hospital, Goyang, Kyungki, Korea; 2 Department of Orthopaedic Surgery, Seoul National University Bundang Hospital, Sungnam, Kyungki, Korea; 3 Medical Research Collaborating Center, Seoul National University Bundang Hospital, Sungnam, Kyungki, Korea; 4 Management Innovation Department QA part, Seoul National University Bundang Hospital, Sungnam, Kyungki, Korea; 5 Department of Orthopaedic Surgery, Seoul National University Children’s Hospital, Seoul, Korea

**Keywords:** Clinical pathway, Supracondylar fracture, Length of hospital stay, Hospital cost

## Abstract

**Background:**

This study was performed to investigate the usefulness of clinical pathway (CP) using an electronic medical record (EMR) in pediatric patients undergoing closed pinning for supracondylar fracture of the humerus, by analyzing the length of hospital stay, hospital cost and satisfaction of the medical teams.

**Methods:**

This before and after comparative study included consecutive children who underwent closed pinning for supracondylar fracture of the humerus since 2009. The pre-CP group consists of 90 patients with the mean age of 5.7 years, and the post-CP group consists of 32 patients with the mean age of 6.2 years. Multidisciplinary work-team developed CP using an EMR system in March 2011. The length of hospital stay was the primary outcome variable, and hospital cost and medical team’s satisfaction score were secondary outcome variables. The non-inferiority test was used to demonstrate the efficiency of the pathway.

**Results:**

The length of hospital stay decreased from 2.9 ± 0.7 days to 2.4 ± 0.7 days by 15.0%, after the implementation of CP, and the lower bound of the 95% CI of the difference (0.14 day) was within the non-inferiority margin of −0.3 days. The hospital cost decreased from 1162.2 ± 236.7 US$ to 1139.8 ± 291.1 US$ by 1.9% and the lower bound of the 95% CI of the difference was −81.3 US$, which did not exceed the non-inferiority margin of −116.2 US$. Therefore, the post-CP group was not inferior compared with the pre-CP group in term of the length of hospital stay and total hospital cost. There was significant increase in the satisfaction score for doctors after implementation of CP (p < 0.001), but, no change in the satisfaction score for nursing staffs (p = 0.793).

**Conclusions:**

The development and implementation of CP, using an EMR, in pediatric patients undergoing closed pinning for supracondylar fracture of the humerus enhances the treatment efficiency by streamlining the treatment process with no increases of the length of the hospital stay and total hospital costs.

## Background

A clinical pathway (CP) is an “optimal sequencing and timing of interventions by physicians, nurses, and other staff for a particular diagnosis or procedure” [1]. Nowadays, a CP is defined as “a complex intervention for the mutual decision making and organization of predictable care for a well-defined group of patients during a well-defined period” [2,3]. CP was initially used in the construction and engineering fields to provide an outline for a given job and its timely completion [4]. Their use in medicine was initially focused on nurses and other non-physician medical staffs [5]. However, with increasing emphasis being placed on the improvement of outcome and efficiency, physicians have also become involved in developing CP. It seeks to streamline costs, improve quality of care, and reduce the length of stay [6]. The pathway details the entire hospital course, by day, for a single procedure or diagnosis.

A CP can provide high quality of medical treatment and minimize the unnecessary medical practice, so it can enhance the treatment efficiency [6]. In addition, CP can improve the patients’ and their guardian’s satisfaction by a predictable medical treatment. CP is thought to improve the communication and cooperation between the medical teams, and eventually improve the satisfaction of the tasks. In the rapidly changing medical environment, the clinical pathway is considered as a method that increases the competitiveness and quality of medical treatment. Numerous studies have demonstrated that a well-designed clinical pathway is an effective means of sustaining the quality, while it can lead to a decrease in postoperative length of stay and hospital charge, such as in colorectal, hepatic, vascular, gynecologic, urologic, and orthopedic procedures [7-13]. However, no study has reported the effectiveness of CP in pediatric orthopedic department.

A supracondylar fracture of the humerus is the most common fracture in children [14]. It is a clinically important fracture in pediatric patients since a displaced fracture may cause serious complications, such as Volkmann ischemic contracture and cubits varus. The mainstream treatment for a displaced supracondylar fracture is a closed reduction and internal fixation using a percutaneous pinning [14]. We think that the development of CP on the treatment for supracondylar fracture of the humerus, especially using an electronic medical record (EMR), can improve the treatment efficiency.

Therefore, we investigated the usefulness of CP, using an EMR, in pediatric patients undergoing closed pinning for supracondylar fracture of the humerus, by analyzing the length of hospital stay, hospital cost and satisfaction of the medical team. We hypothesized that the treatment using CP would be not inferior to the previous treatment regarding the length of hospital stay and hospital cost, and that the implementation of CP could increase the medical team’s satisfaction by standardizing the treatment.

## Methods

This before and after comparative study was approved by the institutional review board at our hospital (SNUBH IRB, B-1105/127-003). Consecutive children, who underwent closed reduction and internal fixation with percutaneous pinning for supracondylar fracture of the humerus, since March 2009, were included in this study. The exclusion criteria were as follows: (1) patients who underwent open reduction and internal fixation; and (2) patients with open fracture or concomitant injury, which requires longer hospital stay. Patients who underwent closed pinning between March 2009 and May 2011, before the implementation of CP, were allocated to the pre-CP group, and patients who underwent closed pinning between June 2011 and May 2012, after the implementation of CP, were allocated to the post-CP group.

From medical records reviews, patients’ age at operation, gender, time from surgery until pin removal, length of hospital stay and complication were obtained. Individual’s hospital cost data were provided from the medical information team and these were subdivided into several categories for comparison: total, non-insurance, room, medication, operating room, anesthesia, laboratory, radiology and materials. These data were compared between the pre-CP group and the post-CP group.

### Development of clinical pathway

We built the multidisciplinary work-team to develop a CP, using an EMR system, for children with supracondylar fracture of the humerus in March 2011. This team involved orthopedic surgeons, nurses for orthopedic ward, quality assurance (QA) team, and a computation team. Our institution, which is the tertiary referral center, achieved the HIMSS (Healthcare Information and Management System Society) Analytics stage 7 for an EMR system [15]. All aspects of patient care were analyzed to the streamline interventions. Orthopedic surgeons and nursing staffs developed an optimal regimen of the treatment processes in patients undergoing closed pinning for supracondylar fracture of the humerus. The final version of the pathway was completed with a consensus by the team members. The contents of the pathway were reviewed and the implementation of pathway was approved by the committee. After the pathway was initiated, it was continuously discussed by the team members so that the pathway could be improved.

A CP consists of a sequence of clinical evaluations, interventions, and standardized care plans with identifiable outcomes. The pathway targeted a 1- to 3-day length of hospital stay, including the preoperative day, the day of the operation and postoperative day. The operation was performed on the day of admission or the following day according to the duration of fasting or the circumstances of the operation room. Patients were recommended to discharge on postoperative day 1 after an observation of immediate postoperative complications. If patients and their guardians want to discharge on the day of the operation or postoperative day 2, 1 day can be added or subtracted from the pathway. Therefore, the pathway can consist of minimum 1 day, and maximum 4 days. If patients needed more than 4 days of hospital stay, they could be dropped out of the pathway, according to the doctor’s judgment (Additional file 1). The education of CP to medical teams, including orthopedic surgeons and nursing staffs responsible for the management of patients, was performed before the implementation of the pathway.

### Implementation of clinical pathway and operative protocols

The CP for pediatric patients with supracondylar fracture of the humerus was implemented using an EMR system, in June 2011. When the indication for surgery was established, the preoperative evaluation, including blood analysis (complete blood count, electrolyte, liver function tests and coagulation studies), electrocardiography, chest x-ray and urinalysis, were performed at the emergency room or the out-patient clinic. If patients had medical history, which showed a risk for general anesthesia, consultation was performed preoperatively. There are two ways to implement CP in EMR system. One is that application of CP was decided at out-patient clinic and ‘Applying the CP’ button was clicked before admission. For this case, application of CP was reconfirmed in EMR system after admission. The other is that ‘Applying the CP’ button was clicked on the ward after patients were admitted to the hospital. Then, the pathway for closed pinning in children with supracondylar fracture of the humerus was selected among various pathways and the implementation of CP was initiated. If it is impossible to execute CP workflow for variation of standard treatment process, the pathway can be discontinued by click the ‘drop-out’ button. Patients and their guardians were informed by the orthopedic resident concerning the perioperative schedules, involving the preoperative evaluation, surgical method, postoperative care, and planned postoperative hospital stay. Lateral pinning technique, using 2 pins, which was found to be more beneficial than the medial and lateral crossed pinning technique on the basis of current evidences [16], were used for the fixation of supracondylar fracture of the humerus. Following the surgery, all patients were immobilized in a long arm cast at least for 3 weeks, according to the radiographic findings. After that period, 2 pins were removed at the out-patients clinic and physical therapy to increase range of motion was not recommended.

### Satisfaction survey of clinical pathway

We developed the questionnaires evaluating the medical team’s satisfaction of the implementation of CP by consensus building session. Satisfaction questionnaires consists of 10 items and the response categories were expressed, using the 5-point Likert scale, of which, the wording was ‘strongly agree’, ‘agree’, ‘neutral’, ‘disagree’, and ‘strongly disagree’ (Additional file 1). Before the implementation of CP, satisfaction questionnaires were completed by 41 medical team members, including 16 medical doctors (orthopedic surgeons and trainees) and 25 nursing staffs for orthopedic wards. Questionnaires were completed again by 35 medical team members including 15 orthopedic surgeons and 20 nursing staffs 1 year after the implementation of CP.

### Statistical methods

The purpose of the study was to test the non-inferiority of the post-CP group to the pre-CP group in terms of the length of hospital stay and hospital cost. The length of hospital stay was the primary outcome variable, and the hospital cost and medical team’s satisfaction score were secondary outcome variables. We had the pre-CP group cohort of 90 patients before this study and their mean hospital stay was 2.9 ± 0.7 days. A mean between-group difference in the hospital stay of 0.5 day was considered clinically significant and assuming a standard deviation of 0.7 day, sample size estimation for a non-inferiority test was performed [17]. A minimum of 120 patients (90 assigned pre-CP group and 30 assigned post-CP group) was required to detect a difference of 0.5 day with 95% power, a one-sided type 1 error rate of 0.05 and allocation ratio of 3:1.

The non-inferiority margin was derived from our historical control group, and it was 10% of the mean values of the length of hospital stay and total hospital cost in pre-CP group. The post-CP group would be judged non-inferior to the pre-CP group if the lower limit of the one-sided 95% CI for the difference in the length of hospital stay and total hospital cost between the two groups was above −0.3 day and −116.2 US$. If non-inferiority was shown, the p value associated with a superiority test was calculated with the Mann–Whitney U test or independent t-test.

Differences in gender, rate of preoperative neurologic deficit and rate of admission on the day of the surgery, between the two groups, were analyzed using a chi-square test or Fisher’s exact test. Mann–Whitney U test or independent t-test was used to analyze the difference in the age at operation, the mean time from the surgery until pin removal, and satisfaction score of the medical team between the two groups. Statistical analyses were conducted using SPSS for Windows (version 18.0; SPSS, Chicago, Illinois).

## Results

Ninety patients in the pre-CP group and 32 patients in the post-CP group were finally included in this study. The mean age of pre-CP group and post-CP group was 5.7 ± 2.4 years and 6.3 ± 3.0 years, respectively. There were 54 male and 36 female in the pre-CP group, and 20 male and 12 female in the post-CP group. Seven of the 90 (7.8%) patients had preoperative neurologic deficit in the pre-CP group, while 2 of the 32 (6.3%) patients did in the post-CP group. There was no statistically significant difference in the age at operation, gender, rate of preoperative neurologic deficit, and the mean time from the surgery until pin removal between the two groups (p = 0.753, 0.804, 0.776, and 0.655, respectively) (Table 1). There was no acute postoperative complication in both groups.

**Table 1:** Comparison of patient demographics and clinical characteristics between pre-CP and post-CP groups

	**Pre-CP group**	**Post-CP group**	**P**
No. of patients	90	32	
Age at operation in years	5.7 ± 2.4 (1.1 to 13.8)	6.2 ± 3.1 (1.4 to 11.7)	0.753
Gender (male/female)	54/36	20/12	0.804
Preoperative neurologic deficit	7 (7.8%)	2 (6.3%)	0.776
Mean time from surgery until pin removal in days	28.7 ± 4.0 (15 to 42)	29.2 ± 5.0 (20 to 42)	0.655

The length of total hospital stay was 2.9 ± 0.7 days in the pre-CP group, and 2.4 ± 0.7 days in the post-CP group. The difference between the two groups was 0.43 days, and the lower bound of the 95% CI of the difference was 0.14 days, which was within the non-inferiority margin of −0.3 days. The total hospital cost was 1162.2 ± 236.7 US$ in the pre-CP group and 1139.8 ± 291.1 US$ in the post-CP group. The difference between the two groups was 36.4 US$, and the lower bound of the 95% CI of the difference was −78.5 US$, which did not exceed the non-inferiority margin of −116.2 US$. Thus, the post-CP group was not inferior compared with the pre-CP group, in term of the length of hospital stay and total hospital cost (Table 2).

**Table 2:** Comparison of the length of hospital stay, hospital cost and medical team’s satisfaction between pre-CP and post-CP groups

**Outcome variables**	**Pre-CP group**	**Post-CP group**	**Mean difference (95% CI)**	**% change**	**P**
Total hospital stay (day)	2.9 ± 0.7 (2 to 5)	2.4 ± 0.7 (1 to 4)	0.43 (0.14 to 0.72)	−15.0%	0.004
Preoperative hospital stay	1.7 ± 0.7 (1 to 4)	1.4 ± 0.5 (1 to 2)	0.28 (0.03 to 0.53)	−16.9%	0.037
Postoperative hospital stay	1.2 ± 0.5 (1 to 3)	1.1 ± 0.4 (0 to 2)	0.14 (−0.01 to 0.31)	−12.3%	0.141
Total hospital cost (US$)	1162.2 ± 236.7	1139.8 ± 291.1	22.4 (−81.3 to 126.1)	−1.9%	0.670
Room	216.3 ± 173.1	217.4 ± 166.7	−1.2 (−71.9 to 69.6)	+0.5%	0.974
Medication	54.1 ± 22.8	55.2 ± 25.7	−1.1 (−10.8 to 8.6)	+2.0%	0.824
Operating room	374.1 ± 62.9	405.6 ± 109.6	−31.6 (−73.7 to 10.5)	+8.4%	0.137
Anesthesia	212.4 ± 34.7	228.1 ± 36.9	−15.7 (−31.9 to 0.4)	+7.4%	0.055
Laboratory	116.0 ± 36.4	83.7 ± 62.3	32.3 (8.3 to 56.2)	−27.8%	0.010
Radiology	80.7 ± 61.9	42.3 ± 22.5	38.4 (15.8 to 60.9)	−47.6%	0.001
Materials	46.0 ± 32.1	70.1 ± 49.4	−24.1 (−39.5 to −8.8)	+52.4%	0.002
Satisfaction score for nursing staff	31.6 ± 4.2 (N = 25)	32.0 ± 5.0 (N = 20)	−0.36 (−3.1 to 2.4)	+1.1%	0.793
Satisfaction score for doctor	36.9 ± 5.5 (N = 16)	45.4 ± 4.3 (N = 15)	−8.5 (−12.1 to −4.8)	+22.9%	<0.001

Using a superiority test, the length of total and preoperative hospital stay decreased significantly after the implementation of CP (p = 0.004 and 0.037, respectively). However, there was no significant difference of postoperative hospital stay between the two groups (p = 0.141). The mean total hospital cost did not decreased after implementation of the CP (p = 0.670). Considering each item of the hospital cost, laboratory and radiologic cost, decreased significantly from 116.0 ± 36.4 and 80.7 ± 61.9 to 83.7 ± 62.3 and 42.3 ± 22.5, respectively (p = 0.010 and 0.001). However, the cost of materials increased significantly from 46.0 ± 32.1 to 70.1 ± 49.4 (p = 0.002) (Table 2).

The satisfaction score for doctors significantly increased from 36.9 ± 5.5 to 45.4 ± 4.3 after implementation of CP (p < 0.001). Of 10 items, doctors were especially satisfied with the items regarding the convenience of the prescription (p < 0.001), convenience of performing the preoperative workup (p < 0.001) and convenience of making a plan for discharge (p < 0.001). However, there was no difference in the satisfaction scores for nursing staff between before and after the implementation of CP (p = 0.793) Of 10 items, the nursing staff were not satisfied with any item after implementation of CP (Table 3).

**Table 3:** Comparison of each item of medical team’s satisfaction questionnaire between pre-CP and post-CP

**Items of satisfaction questionnaire for doctor**	**Pre-CP**	**Post-CP**	**p-value**
Convenience of the prescription	3.8 ± 0.7	4.9 ± 0.4	<0.001
Convenience of performing the preoperative workup	3.7 ± 0.7	4.9 ± 0.4	<0.001
Absence of additional prescription	3.5 ± 1.0	4.5 ± 0.6	0.002
Absence of cancelling the prescription	3.3 ± 0.8	4.3 ± 0.8	0.001
Convenience of the postoperative pain control	3.9 ± 0.7	4.5 ± 0.6	0.012
Convenience of postoperative care	3.8 ± 0.8	4.7 ± 0.5	0.001
Convenience of explaining to the patients and patients’ guardian	3.4 ± 0.5	3.9 ± 0.7	0.016
Convenience of educating junior employees	3.7 ± 0.9	4.3 ± 0.7	0.042
Convenience of transferring the task	3.9 ± 0.9	4.4 ± 0.6	0.118
Convenience of making a plan for discharge	3.9 ± 0.7	4.9 ± 0.3	<0.001
Total	36.9 ± 5.5	45.4 ± 4.3	<0.001
**Items of satisfaction questionnaire for nursing staff**	**Pre-CP**	**Post-CP**	**p-value**
Convenience of providing standardized nurse care	3.3 ± 0.7	3.5 ± 0.7	0.411
Convenience of recognizing the nursing task	3.3 ± 0.7	3.5 ± 0.8	0.332
Absence of additional prescription	2.8 ± 0.9	2.7 ± 1.0	0.600
Absence of cancelling the prescription	2.8 ± 0.7	2.8 ± 0.8	1.000
Enough time to record nursing care	3.0 ± 0.8	3.2 ± 0.8	0.498
Convenience of postoperative pain control	3.1 ± 0.8	3.3 ± 0.9	0.609
Convenience of explaining to the patients and patients’ guardian	3.4 ± 0.7	3.2 ± 1.0	0.519
Convenience of educating junior employees	3.2 ± 0.7	3.3 ± 1.0	0.589
Convenience of transferring the task	3.4 ± 0.7	3.2 ± 0.9	0.227
Convenience of proceeding prompt discharge	3.3 ± 0.7	3.5 ± 0.8	0.583
Total	31.6 ± 4.2	32.0 ± 5.0	0.793

## Discussion

This was the first study investigating the usefulness of CP, using an EMR. This study demonstrated that the application of CP did not increase the length of the hospital stay and total hospital costs, and improved the medical team’s satisfaction in children undergoing closed pinning for supracondylar fracture of the humerus. In this study, the non-inferiority test was used to demonstrate the efficiency of the pathway. Non-inferiority test is used to compare the standard therapy with a new therapy that is expected to have some advantages, such as greater predictability, less side effects, or greater improvement in quality of life [18]. As we do not expect to see a great improvement to the results from the previous treatment, we aim to demonstrate that the implementation of CP is just as good in terms of the length of hospital stay and total hospital cost with non-inferiority test.

There are some limitations to this study. First, this was a mixed retrospective and prospective, comparative study in its design. This study included the historical control group, which could create the potential for bias due to secular trends in the hospital cost. That is, the change in the hospital cost could simply reflect the secular trends and not a direct effect of the CP. A prospective, randomized clinical trial (RCT) is the gold standard for the evaluation of new interventions. However, it is difficult to study using a RCT design because the non-pathway group may be contaminated by the pathway development processes. Second, a primary concern often associated with the reductions in the length of hospital stay is the potential for adverse effects on the patient outcomes, such as an increase in complication rates. In this study, there was no postoperative complication in both groups. However, this study included the patients in the post-CP group with shorter duration of follow-ups than that in the pre-CP group. Therefore, further study on the long-term effects of CP is needed.

A number of studies reported the effectiveness of CP in the orthopedic procedure, including a total knee arthroplasty, total hip arthroplasty and femur neck fracture [19-30]. They investigated the effects of CP on the length of hospital stay, complications, hospital cost, and functional outcomes. Most of them reported that the implementation of CP was associated with reduced length of hospital stay and hospital cost, reduced or unchanged rates of complications, and improvement or no change in the outcomes. However, Mauheran et al. found that the implementation of CP decreased the length of hospital stay, but increased the rate of dislocation, following hip replacement surgery [21]. Roberts et al. showed that the introduction of the pathway for the management of femoral neck fracture in older patients was associated with the improved clinical outcomes, but longer hospital stay and increased use of occupational therapy [30]. Barbieri et al. performed the meta-analysis on the effects of CP in the hip and knee joint replacement. They concluded that CP could significantly improve the quality of care in terms of postoperative complications, length of hospital stay and hospital costs. However, it was not possible to conclude that the implementation of CP was a cost-effective process, because none of the included studies analyzed the cost of the development and implementation of the pathways [31].

The length of total hospital stay in the post-CP group was not longer than that in the pre-CP group, as expected. Furthermore, there was a significant reduction of the total hospital stay, which consisted of preoperative and postoperative hospital stay. Discharge planning was an important aspect of the pathway’s development and implementation. Our pathway included the planned discharge the day after the surgery. However, there was no difference of postoperative hospital stay between the two groups (p = 0.141). Preoperative hospital stay showed a significant reduction (p = 0.037), and might be affected more than postoperative hospital stay by the implementation of CP. It implied that our pathway could streamline the preoperative evaluation process.

This study was performed outside the United States, which has a national health insurance system, which is sponsored by a government agency. It is a universal system that covers approximately 95% of the population in this country. Therefore, the hospital cost in our country is relatively less expensive than that in the United States. In this study, the total hospital cost was not increased after the implementation of the CP. We expected that the total hospital cost would decrease because of shorter lengths of hospital stay. Using a superiority test, there was no reduction of the total hospital cost (p = 0.531). We think, the fact that the inflation of medical insurance cost was not considered in this study, might result in this finding. This study showed a significant increase of the material cost after the implementation of CP (p = 0.002). In addition, the cost of anesthesia was not changed after the implementation of CP. The reason was that no modification of and anesthesia regimen was introduced during the study period. On the other hand, there was a significant reduction of laboratory and radiologic cost. It implied that the implementation of CP could eliminate the unnecessary expenses regarding the laboratory and radiologic examinations.

Three hospitals, including our institution, are affiliated with the same university, and all orthopedic residents rotate among the three hospitals during the course of their residency. They seemed to have difficulty in managing patients due to different treatment policy of each hospital. Therefore, the implementation of the pathway might increase the doctors’ satisfaction score by establishing uniformity of management in this study. The scores of all items increased significantly except the item regarding the convenience of transferring the task. However, none of the items were scored as satisfactory by the nursing staff after the implementation of CP, unlike the doctors’ response. Although it was one of the goals of the pathway to improve patient satisfaction with care by educating patients and their families about the plan of care [6], our study did not include those as the outcome variable. Further study is required on the effectiveness of CP in terms of patients’ and their guardian’s satisfaction.

The system of diagnosis-related groups (DRGs), which classifies inpatients into clinically meaningful homogeneous classes, based on the expected costs of the treatment, has been used in many countries. Major goals for implementation of DRG has been reducing the health care expenditures and cost control by setting hospitalization payment for all payers at a fixed DRG-rate per admission. Accurate costs of treatment is fundamental to the operation of institutions where prospective payments are made in accordance with diagnosis-related groups (DRGs), standardized lengths of stay and fixed reimbursement for care. Therefore, the development of the CP could be the base of the development of DRG in orthopedic procedures.

In this study, the development and implementation of CP provided treatment efficiency, in terms of the length of the hospital stay, hospital cost and doctors’ satisfaction. However, a CP is a complex or multicomponent intervention that is more than just a document providing efficiency. The CP should be developed and implemented considering a patient-focus care paradigm which improves risk-adjusted patient outcomes, safety and satisfaction, optimizing the use of resources and facilitation of communication among team members and with patients, as well as considering the coordination of the care process by coordinating the roles, and sequencing the activities of the multidisciplinary care team and patients.

## Conclusions

The development and implementation of CP, using an EMR, in pediatric patients undergoing closed pinning for supracondylar fracture of the humerus enhances the treatment efficiency by streamlining the treatment process with no increases of the length of the hospital stay and total hospital costs. This study could be the basis for further development of CPs for other orthopedic procedures and the implementation of DRG in the future.

### Consent

Written informed consent was obtained from the patient's guardian/parent/next of kin for the publication of this report and any accompanying images.

## Abbreviations

CP: Clinical pathway; EMR: Electronic medical record; DRG: Diagnosis-related groups.

## Competing interests

The authors declare that they have no competing interests.

## Authors’ contributions

CYC, IHC and MSP participated in the design of this study. KHS, KML, SYL and JHL participated in data acquisition and collection. KHS, MSP, TJC and WJY analyzed the data. KHS, SMP and MSP drafted the manuscript. SYA and KHS performed the statistical analysis. All authors participated in data interpretation, and read and approved the final manuscript.

## Pre-publication history

The pre-publication history for this paper can be accessed here:

http://www.biomedcentral.com/1472-6947/13/87/prepub

## Supplementary Material

Additional file 1 Details of the order set in clinical pathway and Satisfaction questionnaire for doctors and nursing staffs. Click here for file
