# Supplementary material for: Application of clinical pathway using electronic medical record system in pediatric patients with supracondylar fracture of the humerus: a before and after comparative study
Source: BMC Med Inform Decis Mak. 2013 Aug 11;13:87. doi: 10.1186/1472-6947-13-87 (PMC3751125; doi:10.1186/1472-6947-13-87)
Supplement: Additional file 1 — Details of the order set in clinical pathway and Satisfaction questionnaire for doctors and nursing staffs. [file 1472-6947-13-87-S1.doc]

| Appendix 1. Details of the order set in clinical pathway for closed pinning in children with supracondylar fracture of the humerus. | | | | | |
| --- | --- | --- | --- | --- | --- |
|  | | Management | Diet | Injection or medication | Laboratory or radiographic exam |
| First day  (preoperative day) | | Check vital sign q 8hr  Bed rest  Skin preparation  Get operation permission | NRD->  Midnight NPO | Cefazolin SC for AST  Prn) Ketorolac tromethamine 0.5-1mg/kg plus normal saline 50ml IV  Prn) Acetaminophen syrup 10-15mg/kg PO  Prn) Pethidine hydrochloride 0.5-2mg/kg plus normal saline 50ml IV | Chest x-ray, Electrocardiography  ABO/Rh Type & Antibody screening  Admission Panel, Electrolyte panel  Coagulation Panel  Anti-HBs, HBsAg, VDRL  Urinalysis with microscopic exam |
|  | |  |  |  |  |
| Second day  (operation day) | Preop | Check vital sign q 8hr  Bed rest  Check premedication  Send to operation room | NPO | Hartmann solution 10ml/kg/hr IV |  |
|  | Intraop |  |  | Cefazolin 20-40mg/kg/day IV at operation room | Right or Left elbow AP/Lat at operation room |
|  | postop | Check vital sign q 15min till stable, then q 1hr (*4)  -> q 8hr  Bed rest  Arm elevation | NPO till gas out, then SOW ->  SFD -> SBD | Dextrose 5% NaK 10ml/kg/hr IV  Prn) Ketorolac tromethamine 0.5-1mg/kg plus normal saline 50ml IV  Prn) Acetaminophen syrup 10-15mg/kg PO  Prn) Pethidine hydrochloride 0.5-2mg/kg plus normal saline 50ml IV |  |
|  | |  |  |  |  |
| Third day  (postoperative day) | | Check vital sign q 8hr  Ward ambulation  Arm elevation  Discharge home  Follow-up at out-patient clinic 1week postoperative | NRD | Prn) Ketorolac tromethamine 0.5-1mg/kg plus normal saline 50ml IV  Prn) Acetaminophen syrup 10-15mg/kg PO  Prn) Pethidine hydrochloride 0.5-2mg/kg plus normal saline 50ml IV  Acetaminophen syrup 10-15mg/kg PO tid* 5 days (discharge medication) | Right or Left elbow AP/Lat at out-patient clinic 1 week postoperative |
| NRD, normal regular diet; SOW, sips of water; SFD, soft fluid diet; SBD, soft blend diet  This order set consists of 3-day length of hospital stay as above. If needed, the pathway could be reduced to minimum 1-day set or could be lengthened by 4 day-set of maximum.  A single dose of antibiotics injection within 1 hour prior to surgery was recommended.  Intravenous patient-controlled analgesia (IV PCA) was used for postoperative pain control in case of parents need. If pain relief was insufficient, intravenous ketorolac tromethamine was given, followed by oral acetaminophen syrup and intravenous pethidine hydrochloride. | | | | | |

Appendix 2. Satisfaction questionnaire about the current management system for doctors and nursing staffs

Questionnaire (for doctor)

This is a questionnaire of the level of satisfaction with current management system for pediatric patients with supracondylar fracture of the humerus. Please circle the number representing the appropriate answer.

1. You are satisfied with a convenience of the prescription.

| 5 | 4 | 3 | 2 | 1 |
| --- | --- | --- | --- | --- |
| Strongly Agree | Agree | Neutral | Disagree | Strongly Disagree |

2. You are satisfied with a convenience of performing the preoperative workup.

| 5 | 4 | 3 | 2 | 1 |
| --- | --- | --- | --- | --- |
| Strongly Agree | Agree | Neutral | Disagree | Strongly Disagree |

3. You are satisfied with the absence of additional prescription.

| 5 | 4 | 3 | 2 | 1 |
| --- | --- | --- | --- | --- |
| Strongly Agree | Agree | Neutral | Disagree | Strongly Disagree |

4. You are satisfied with the absence of cancelling the prescription.

| 5 | 4 | 3 | 2 | 1 |
| --- | --- | --- | --- | --- |
| Strongly Agree | Agree | Neutral | Disagree | Strongly Disagree |

5. You are satisfied with a convenience of the postoperative pain control.

| 5 | 4 | 3 | 2 | 1 |
| --- | --- | --- | --- | --- |
| Strongly Agree | Agree | Neutral | Disagree | Strongly Disagree |

6. You are satisfied with a convenience of postoperative care.

| 5 | 4 | 3 | 2 | 1 |
| --- | --- | --- | --- | --- |
| Strongly Agree | Agree | Neutral | Disagree | Strongly Disagree |

7. You are satisfied with a convenience of explaining to the patients and patients’ guardian.

| 5 | 4 | 3 | 2 | 1 |
| --- | --- | --- | --- | --- |
| Strongly Agree | Agree | Neutral | Disagree | Strongly Disagree |

8. You are satisfied with a convenience of educating junior employees.

| 5 | 4 | 3 | 2 | 1 |
| --- | --- | --- | --- | --- |
| Strongly Agree | Agree | Neutral | Disagree | Strongly Disagree |

9. You are satisfied with a convenience of transferring the task.

| 5 | 4 | 3 | 2 | 1 |
| --- | --- | --- | --- | --- |
| Strongly Agree | Agree | Neutral | Disagree | Strongly Disagree |

10. You are satisfied with a convenience of making a plan for discharge.

| 5 | 4 | 3 | 2 | 1 |
| --- | --- | --- | --- | --- |
| Strongly Agree | Agree | Neutral | Disagree | Strongly Disagree |

Questionnaire (for nursing staff)

This is a questionnaire of the level of satisfaction with current management system for pediatric patients with supracondylar fracture of the humerus. Please circle the number representing the appropriate answer.

1. You are satisfied with a convenience of providing standardized nurse care.

| 5 | 4 | 3 | 2 | 1 |
| --- | --- | --- | --- | --- |
| Strongly Agree | Agree | Neutral | Disagree | Strongly Disagree |

2. You are satisfied with a convenience of recognizing the nursing task.

| 5 | 4 | 3 | 2 | 1 |
| --- | --- | --- | --- | --- |
| Strongly Agree | Agree | Neutral | Disagree | Strongly Disagree |

3. You are satisfied with the absence of additional prescription.

| 5 | 4 | 3 | 2 | 1 |
| --- | --- | --- | --- | --- |
| Strongly Agree | Agree | Neutral | Disagree | Strongly Disagree |

4. You are satisfied with the absence of cancelling the prescription.

| 5 | 4 | 3 | 2 | 1 |
| --- | --- | --- | --- | --- |
| Strongly Agree | Agree | Neutral | Disagree | Strongly Disagree |

5. You are satisfied with a enough time to record nursing care.

| 5 | 4 | 3 | 2 | 1 |
| --- | --- | --- | --- | --- |
| Strongly Agree | Agree | Neutral | Disagree | Strongly Disagree |

6. You are satisfied with a convenience of postoperative pain control.

| 5 | 4 | 3 | 2 | 1 |
| --- | --- | --- | --- | --- |
| Strongly Agree | Agree | Neutral | Disagree | Strongly Disagree |

7. You are satisfied with a convenience of explaining to patients and the patients’ guardian.

| 5 | 4 | 3 | 2 | 1 |
| --- | --- | --- | --- | --- |
| Strongly Agree | Agree | Neutral | Disagree | Strongly Disagree |

8. You are satisfied with a convenience of educating junior employees?

| 5 | 4 | 3 | 2 | 1 |
| --- | --- | --- | --- | --- |
| Strongly Agree | Agree | Neutral | Disagree | Strongly Disagree |

9. You are satisfied with a convenience of transferring the task?

| 5 | 4 | 3 | 2 | 1 |
| --- | --- | --- | --- | --- |
| Strongly Agree | Agree | Neutral | Disagree | Strongly Disagree |

10. You are satisfied with a convenience of proceeding prompt discharge.

| 5 | 4 | 3 | 2 | 1 |
| --- | --- | --- | --- | --- |
| Strongly Agree | Agree | Neutral | Disagree | Strongly Disagree |
